# The importance of the smallest effect size of interest in expert witness testimony on alcohol and memory

**DOI:** 10.3389/fpsyg.2022.980533

**Published:** 2022-12-05

**Authors:** Henry Otgaar, Paul Riesthuis, Johannes G. Ramaekers, Maryanne Garry, Lilian Kloft

**Affiliations:** ^1^Faculty of Law and Criminology, KU Leuven, Leuven, Belgium; ^2^Faculty of Psychology and Neuroscience, Maastricht University, Maastricht, Netherlands; ^3^School of Psychology, The University of Waikato, Hamilton, New Zealand

**Keywords:** alcohol, memory, eyewitness, false memory, reliability, smallest effect size of interest

## Abstract

Memory experts are sometimes asked to evaluate the validity of accounts of witnesses, victims, or suspects. In some of these cases, they are asked what effect alcohol has on the validity of such accounts. In this article, we offer a guide on what expert witnesses can reliably say about how alcohol affects memory. We do so by resorting to effect sizes from previous studies and meta-analytic work, and address this novel question: Are these effect sizes meaningful in legal cases? More specifically, we argue that any determination of whether individual studies about alcohol and memory are practically relevant for legal cases, scientists must focus on the smallest effect size of interest. We make the case that a decrease or increase of only 1 detail, especially an incorrect detail, should be regarded as the smallest effect size of interest in this line of research. In line with this idea, we show that effect sizes in the alcohol and memory literature are often larger than this smallest effect size of interest. This finding is important because it implies that alcohol often exerts a practically relevant and meaningful detrimental effect on the reporting of both correct and incorrect details, which in turn negatively affects the validity of witness testimony.

## Introduction

Triers of fact face immense challenges when they have to render legal decisions in cases in which the primary evidence consists of testimony from alleged victims, witnesses or suspects. The reason is forthright. Although our memories are generally accurate, numerous factors can distort our memories, and lead to invalid testimony^1^ (Wixted et al., 2018; Otgaar et al., 2022). The major bottleneck with invalid statements is they can lead to wrongful convictions—and hence, miscarriages of justices (Howe and Knott, 2015). It is therefore vital to identify factors that potentially undermine memory.

One factor that has been heralded as inherently problematic is alcohol (Jores et al., 2019). Crimes are often perpetrated when victims, witnesses or suspects are intoxicated. Court rulings^2^ involving the consumption of alcohol (or other drugs) represent about 1.2–4.3% of all legal cases (Kloft et al., 2021). But these percentages are even higher when we focus on violent crimes. For instance, statistics for violent crimes, such as homicide, show that 25 to 78% of suspects and 24 to 72% of victims are under the influence of drugs. In many of these cases, intoxicated witnesses, victims, and suspects talk about their experiences with, for example, the police. Besides the fact that alcohol exerts a huge toll in many crimes, a critical question is whether intoxicated victims, witnesses, and/or suspects can provide valid statements.

One of the main problems in legal cases including alcohol is that triers of fact need to decide the extent to which intoxicated people provided a valid account of the alleged events to, for example, the police. An extra dimension to this problem is that legal professionals often view alcohol as a factor impeding the credibility of testimony, thereby reducing the likelihood for a conviction (Kassin et al., 2001; Evans and Schreiber Compo, 2010; Monds et al., 2022).

Legal professionals sometimes ask the assistance of memory experts to testify about the effect of alcohol on the validity of memory and testimony. In the current article, we describe what memory experts can safely say about how alcohol affects the validity of eyewitness testimony when they provide expert testimony in court. In doing so, we argue that it is imperative to look at the effect sizes detected in empirical research on alcohol and memory, and decide what the smallest effect size of interest is (Lakens, 2017). Then, we re-analyze data from a recent meta-analysis on alcohol and memory (Jores et al., 2019) and interpret our new analyses refracted through our proposed smallest effect size of interest.

## Validity of testimony

When legal professionals and memory experts refer to the “validity” of testimony of victims, witnesses, and suspects, validity is regularly regarded as a testimony representing an accurate depiction of what was experienced. Hence, the term *accuracy* is often applied in this context as well. Importantly, when we talk about the validity of testimony, we refer to whether the details reported in the testimony refer to truly experienced details. When victims, witnesses, and suspects provide testimony, they generally have given a statement (or statements) on what purportedly occurred during an event. Of course, assessing the degree to which statements are accurate is arduous because the ground truth of many crimes is unknown. Therefore, scientists frequently focus on other related concepts, such as the *completeness* and *consistency* of statements. Completeness describes the number of (correct) details in a statement (where ground truth thus is also needed), whereas consistency describes the reporting of similar details at repeated occasions. For instance, a statement is highly complete when it contains many (correct) details and a statement is inconsistent when a certain detail is reported in an initial statement, but is changed at follow-up statements. Although accuracy and completeness are difficult to determine in legal cases, they can be studied in the psychological laboratory. In these studies, scientists ask participants to view some stimuli, or let them engage in an interactive event. Later, participants provide statements about what they experienced. Because these experimental designs allow scientists to know the objective truth about what truly unfolded during the event, accuracy and completeness^3^ can be investigated (Smeets et al., 2004).

Although inconsistent statements are frequently regarded as inaccurate statements, at a statement level, accuracy and consistency are not strongly related to each other. For example, Brewer et al. (1999) presented participants with a video of a mock bank robbery and then interviewed them twice (2-weeks interval) about the event, and found that consistency was not a strong predictor for accuracy. Moreover, in two studies, Smeets et al. (2004) provided participants with violent movie fragments, and asked them to report, on two occasions, what they still remembered concerning these fragments. Here too, inconsistencies were not related to inaccuracies in statements. Furthermore, completeness was only moderately, positively, related to accuracy.

Although inconsistent statements do not imply inaccurate statements, inconsistent statements can also arise from contradictions between statements. For example, a witness might report at the first interview that a robber carried a gun while reporting that he had a knife at the second interview. Contradictions clearly indicate inaccuracies in at least one version of events. Having said that, repeated interviews do not necessarily lead to contradictory statements. Specifically, when repeated interviews are well conducted, follow up interviews might lead to new details that are accurate, yet inconsistent with earlier statements (Goodman and Quas, 2008). Also, although Smeets et al. (2004) showed that completeness was modestly related to accuracy, even highly inaccurate statements can *appear* to be very detailed and complete. For example, even entirely fictitious autobiographical memories can have a high level of details (see, for a review, Scoboria et al., 2017).

In general, then, when considering the validity of testimony, memory experts and other expert witnesses also consider other aspects related to the validity of testimony, such as consistency and completeness. We now turn to research on the effect of alcohol on the validity of testimony.

## Alcohol and memory

A variety of experiments have been conducted on the effect of alcohol on memory for witnesses and perpetrators. For example, in one of the first studies, Yuille and Tollestrup (1990) had participants watch a staged theft. Some were under the influence of alcohol during encoding (mean blood alcohol content: 0.10 ml). Furthermore, some participants were interviewed immediately about the event, and all were interviewed 1 week later. Alcohol reduced the amount of details that were reported at the immediate interviews and also reduced accuracy at the delayed interview.

Other studies have not detected any effect of alcohol on the validity of eyewitness statements. For example, in a study by Schreiber Compo et al. (2011), intoxicated (mean BAC: 0.07 g/210 l), placebo, and sober eyewitnesses watched a staged theft, received some false information about the event, and were then immediately interviewed about the theft. Alcohol intoxication did not have any notable effect on the number of accurate and inaccurate details. Instead of focusing solely on eyewitness memory, studies have also considered the influence of alcohol on perpetrator memory. For example, in van Oorsouw et al. (2012) field study, participants were approached in bars and were asked to view a mock crime video from a perpetrator perspective. After 3–5 days, participants received a free recall and a cued recall test. In general, intoxicated participants (high intoxicated: *M*_BAC_ = 0.16%, moderately intoxicated: *M*_BAC_ = 0.06%) were less complete in their memory reports in free recall scores and also less accurate in their cued recall scores than sober participants.

Overall, applied studies examining alcohol-memory effects have used slightly different procedures (for instance, field versus experimental studies) and sometimes generated discrepant results (memory-undermining or no memory undermining effects of alcohol; e.g., LaRooy et al., 2013; Hagsand et al., 2017). To address this disparity in the literature, a recent meta-analysis combined all studies and examined the effects of alcohol intoxication on recall (Jores et al., 2019). The main conclusions were as follows. First, alcohol intoxication statistically *significantly* reduced the number of correct details but did not notably (statistically significantly) affect the number of incorrect details. Second, the authors looked at whether memory-undermining effects of alcohol were moderated by several key factors intoxication level, memory test (cued versus free recall). For example, alcohol exerted larger reductions of correct details on cued than free recall.^4^

What are we to make of these findings? The authors themselves said their “findings have important implications for the legal system because, contrary to commonly held views in the legal system that intoxicated witnesses are unreliable, our results suggest that while intoxicated compared with sober witnesses provide testimony that is less complete, their testimony is no more likely to contain inaccurate details (p. 342).” This claim was based, in part, on the observed effect sizes detected in their meta-analysis, in which alcohol wielded a “significant and moderate sized effect” on free recall of correct details (p. 334).

The importance of the use and interpretation of effect sizes in research on alcohol and memory has already been described by Schreiber Compo et al. (2011) who wrote that “whether possible differences among alcohol, placebo, and sober witnesses will be considered substantial and thus relevant to law enforcement, expert witnesses and policy makers will likely depend on the effect size of any given difference in quantity and quality of witness testimony” (p. 2). A crucial question, then, is how memory experts can apply these observed effect sizes when they testify in the legal arena about the effects of alcohol on memory. But memory experts can apply effect sizes only when it is clear what the smallest effect size of interest is.

## Effect sizes and memory research

Psychological scientists frequently calculate effect sizes to denote the magnitude of a certain phenomenon—yet there is often a disconnection between observed effect sizes and practical implications. Even if they make this connection, scientists often use benchmarks to denote the practical significance of their work (Anvari et al., 2022). For instance, terms such as *small*, *medium*, and *large* effect sizes are commonly applied but they are rarely put into a specific context. These terms are often attributed to Cohen (1988), but even he bemoaned the casual translation of effect sizes into t-shirt sizes, saying “The terms ‘small’, ‘medium’, and ‘large’ are relative, not only to each other, but also to the area of behavioral science or even more particularly to the specific content and research method being employed in any given investigation” (p. 25). Put another way, without providing context, these terms are in themselves meaningless.

Consider, for example, a scientist who reports that a certain type of drug leads to a statistically significant increase in memory errors, constituting a medium effect size of *d* = 0.5, according to Cohen (1988) conventional benchmarks. How should we interpret this finding? Should we worry, perhaps, that this drug tends to negatively affect memory? Do we even agree what it means to show a medium effect size in the field of memory? Might we, for instance, connect the idea to a certain number of memory errors detected in a controlled study? But let us not confine this discussion to just the scientific community. If we go into the community, such as serving as memory expert in court, what would we say about this drug and its tendency to result in, say, inaccurate statements? Clearly, we need some sensical threshold that makes sense in the context where we use it. One way to accomplish these aims is to decide on the smallest effect size of interest (Lakens, 2014).

In short, the smallest effect size of interest is the smallest effect that (1) researchers personally care about, (2) is theoretically interesting, or (3) has practical relevance (Anvari and Lakens, 2021). Different methods exist to establish a smallest effect size of interest in any domain of psychology. For example, anchor-based methods such as the minimal clinically important difference refer to substantiating the smallest difference by which a patient subjectively notices improvement (or worsening; Jaeschke et al., 1989).

To establish the smallest effect size of interest one can also question psychologists about what they regard as the smallest effect size of interest in their domain. In the first study of this kind in the field of memory, Riesthuis et al. (2022) surveyed several memory researchers (*N* = 41) to obtain the smallest effect size of interest for false memory research. Participants read several scenarios containing the method and procedure of frequently-used false memory paradigms, such as an experiment on the effect of therapy on the formation of false memories. For each scenario, participants determined the smallest effect size of interest for theoretical and practical ends. Interestingly, there was no evident agreement among memory scientists when it came to the smallest effect size of interest in false memory research. Nonetheless, these scientists tended to prefer smaller effect sizes of interest or any statistically significant effect (that is, any effect leading to a *p* < 0.05), especially for theoretical ends.

As sound as these ideas might be for the advancement of theory and identification of mechanisms, we suggest it does not work when it comes to rendering practical assistance in fields, such as in court. Taken this issue and the findings by Riesthuis et al. (2022) into account, our proposal for the smallest effect size of interest in the area of applied memory research is that any factor—such as alcohol—that causes a gain or loss of even *one* correct or incorrect reported detail can be of significant practical importance (see Otgaar et al., 2022). One forgotten detail, or one incorrectly reported detail, could result in severe consequences in the courtroom. For example, an eyewitness who falsely remembers that an innocent bystander was the culprit might make a false accusation. Also, an eyewitness who forgets the color of the clothing worn by the perpetrator might make it more challenging to undertake a criminal investigation (see for example Strange et al., 2014). In short, to the extent that research shows that alcohol intoxication leads to the forgetting of 1 (or more) detail or the reporting of 1 (or more) incorrect detail, these are findings of high interest for the courtroom. Also, as one detail can potentially have negative consequences, we argue that our smallest effect size of interest is any detail that is forgotten or (mis)remembered. Of course, our choice for the smallest effect size of interest reflects some arbitrariness and is open for discussion. Nonetheless, scholars have made the important point that the practical relevance of the smallest effect size of interest hinges on—amongst other aspects—on a cost–benefit analysis of the chosen effect size (Anvari et al., 2022). In the field of applied memory research, there are good reasons for the case that the forgetting of even one correct detail, and (or) the reporting of one incorrect detail, is an important effect size of interest. Failing to report one detail, or falsely remembering one detail, sounds trivial—but when that detail is the name of the perpetrator, an inaccurate description of his appearance, or which weapon he brandished— are all examples of details that, when wrong, can lead to false accusations and potentially even wrongful convictions. Therefore, our choice of this smallest effect size of interest is not only non-trivial, but based on a strict and conservative cost–benefit analysis, such as the goal of avoiding wrongful convictions while actual perpetrators roam free committing more crimes. Furthermore, research shows that detailed testimonies are regarded as more credible in court which means that when such testimonies contain an incorrect detail, the credibility is unduly based on inaccuracy (e.g., Spellman and Tenney, 2010). However, it is difficult to indicate which details might impact legal decisions more than others. That is, in certain cases details that are otherwise considered irrelevant such as the color of a jacket, can be crucial in others as seen in the case of Anna Lindh (Granhag et al., 2013). The case of Anna Lindh illustrates the difficulty to pinpoint *a priori* which details are more relevant for legal decisions as they are dependent on the individual case characteristics. In short, one possible benefit of adopting such a strict and cautious smallest effect size is that false accusations and wrongful convictions can be prevented.

Of course, memory scientists might decide that in other circumstances, more liberal “smallest effect sizes of interest” are warranted—say, forgetting of 5 details, misremembrance of 5 details. However, the downside of such a liberal decision is that when a witness falsely remembers that the culprit wore a blue jacket and red trousers (i.e., 2 incorrect details), a wrong person could be apprehended potentially leading to false accusations. Our strict smallest effect of size is therefore also related to the adage of the Blackstone Ratio that dictates that it is better to have 10 guilty persons escape than have 1 innocent person be imprisoned. Alternatively, scholars might argue that adopting a strict criterion will discredit real victims of sexual abuse who were intoxicated at the time of the crime. Therefore, it is important to continue discussions on the arguments behind choosing smallest effect sizes of interest.

## The smallest effect size of interest in the context of alcohol and memory: A re-analysis of meta-analytic data

Now that we have made a case about which effect size potentially is practically relevant in applied memory research, with this knowledge, we re-analysed the data from the meta-analysis of Jores et al. (2019).^5^ The ultimate goal was to obtain the raw mean differences of how alcohol impacted the validity of testimony in these studies and examine them in light of our smallest effect size of interest. Since the meta-analysis reported only standardized effect sizes (Hedges’ *g*), we specifically investigated and calculated the raw mean differences between intoxicated and non-intoxicated people.

Our first exercise was to examine one of the main findings of the meta-analysis, in which the authors showed that alcohol intoxication led to a reduction of correct details with an effect size of 0.40 (Hedges’ *g*; 95% CI [0.21, 0.59], *p* < 0.001). This effect size amounted to a mean difference of 3.05 details (*SD* = 9.84). If we compare this number with our smallest effect size of interest, it is obvious that the observed effect size in the meta-analysis is practically relevant. Even more, the effect size in the meta-analysis is about three times the smallest effect size of interest, a finding that suggests that alcohol intoxication is associated with problems for the completeness of memory to a concerning level of practical significance.^6^ In line with this conclusion, we also conducted an equivalence test using JASP (Lakens, 2017) in which we compared the raw mean difference with an equivalence region of −1 to 1. This region stands for our smallest effect size of interest. Equivalence tests are recommended because scientists often want to test for the presence *and* absence of an effect. Equivalence tests can be used to determine, then, whether an observed effect is too small to care about given the smallest effect size of interest, even if statistically significant (Lakens, 2017). Equivalence tests are conducted to examine whether a certain effect is equivalent to a specified smallest effect size of interest. If no equivalence is detected, the observed difference can be considered meaningful. The conducted equivalence test did not find a statistically significant effect (*t*(94) = 0.43, *p* = 0.67) implying that the mean differences are not equivalent to our smallest effect size of interest. This suggests that the observed mean difference is indeed meaningful and practically relevant.

An alternative way to interpret the main effect of the meta-analysis is to use the common language effect size (CLES; McGraw and Wong, 1992). Using the CLES, an effect size of 0.40 (Cohen’s *d*) indicates that there is a 61.1% chance that a person picked at random from the control group will have a higher score than a person picked at random from the alcohol group (McGraw and Wong, 1992). Moreover, with this effect size (Cohen’s *d* = 0.4), 65.5% of the control group will be above the alcohol group (U_3_; Grice and Barrett, 2014).^7^

Our second analysis concerned the effect of alcohol on the reporting of incorrect details. Interestingly, although the meta-analysis (Jores et al., 2019) showed that alcohol intoxication did not have a statistically *significant* effect on the reporting of incorrect details (Hedges’ *g* = −0.08, 95% CI [−0.21, 0.05], *p* = 0.23), the raw mean difference amounted to 1.37 details (*SD* = 3.13) implying that alcohol increased the reporting of incorrect details. Strictly speaking, this difference is a bit higher than our proposed smallest effect size of interest, which in turn implies that this difference might be of practical relevance. Indeed, an equivalence test showed that this raw mean difference was not equivalent to our smallest effect size of interest, implying practical relevance and meaningfulness (*t*(46) = −0.74, *p* = 0.47). Nonetheless, caution is warranted in stressing practical relevance here because it might be the case that the studies of the meta-analysis were not sufficiently powered to detect a range of effect sizes, such as our smallest effect size of interest.

We also examined whether this difference might differ for free and cued recall. For free recall, we detected a raw mean difference of 0.38 (*SD* = 2.19). This small difference is in line with the Jores et al. (2019) who argued that intoxicated witnesses are not necessarily invalid because alcohol does not seem to significantly increase the reporting of incorrect details during free recall. Interestingly, although the raw mean difference was in the region of the smallest effect size of interest, they were not statistically equivalent (*t*(30) = −0.21, *p* = 0.84). The reason for this is that although the raw mean difference fell within this region, there was a huge variation in the data outside this region as revealed by the 90% CI [−3.51, 2.75].

For incorrect details at cued recall, we obtained a mean difference of 3.60 details (*SD* = 4.14). This number is even higher than the correct detail data and clearly indicates that at least for cued recall, alcohol intoxication does lead to invalid testimony which is also practically relevant (see also Table 1). Here too, an equivalence test showed that this raw mean difference was not equivalent to our specific smallest effect size of interest (*t*(12) = −0.83, *p* = 0.42). To further examine whether this raw mean difference does lead to a statistically significant meta-analytic finding, we re-ran the meta-analysis of Jores et al. (2019).

**Table 1:** Practical relevance of the detected mean differences.

	Mean difference	Practically relevant: Yes versus no	Hedges’ *g* 95% CI in brackets
Free recall - correct	3.03	Yes	0.35 (0.14, 0.56)
Cued recall - correct	3.10	Yes	0.52 (0.26, 0.77)
Free recall - incorrect	0.38	No	0.07 (−0.07,0.20)
Cued recall - incorrect	3.60	Yes	−0.37 (−0.57, −0.17)

With practical relevance, we refer to whether the mean difference is higher than the proposed smallest effect size of interest: Yes means that the mean difference is higher than the smallest effect size of interest, no means that it is lower. All green cells refer to statistically significant meta-analytic effect sizes, red cells refer to statistically non-significant meta-analytic effect sizes.

**Figure 1:**
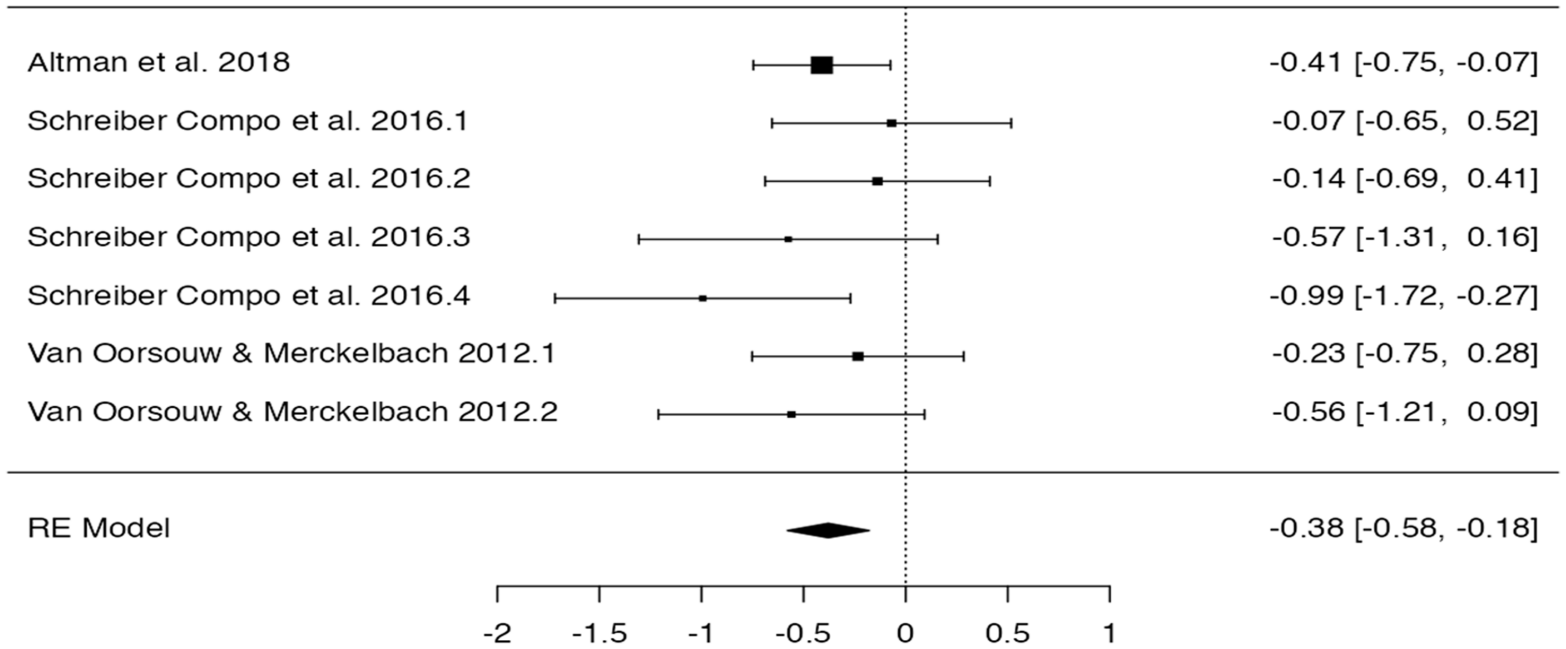
Forest plot concerning alcohol intoxication and incorrect details (cued recall).

Specifically, using jamovi (2.3.13.0), we included the cued recall data on incorrect details and performed a meta-analysis with a Hunter-Schmidt model estimator. We chose this model estimator (and jamovi) because it was also used in Jores et al. (2019). Interestingly, our meta-analysis showed that alcohol intoxication led to a statistically significant increase of incorrect details (*p* < 0.001; see Table 1 and Figure 1 for the forest plot). So, our detected mean difference of 3.60 details was related to a statistically significant effect of alcohol intoxication on the reporting of incorrect details concerning the cued recall data. It is important to stress again here that even though effects can be statistically significant, this does not mean that they are immediately practically relevant. When sample sizes are large enough, effects might become statistically significant, although the effects are in themselves trivial (see also Montes et al., 2022). It is crucial to point out that small effects might sometimes also have practical relevance (Funder and Ozer, 2019). Take for instance the example of aspirin consumption on heart attack occurrences wherein they found that a conventional small effect size (Pearson *r* = 0.03) meant that 85 heart attacks were prevented. Again, we stress the importance of contextualizing any observed effects and establish the smallest effect size of interest for the specific area of research.

As a side note, it is important to state that the estimates and 95% confidence intervals of the meta-analytic data varied widely and did not account for the non-independency among effect sizes (Cheung, 2019). The variation among the estimated effect sizes can be due to a multitude of factors such as that the research and study designs, measurement instruments, and stimuli differed between studies that were included in the meta-analysis, while the confidence intervals are dependent on sample size and the variation. In general, the smaller these confidence intervals, the more accurate the estimates (Cumming, 2008). Moreover, multiple effect sizes of the same sample were present which can alter the mean effect size (Cheung, 2019). However, our main focus of the present article was to re-analyze the reported findings in terms of practical relevance regarding the SESOI and not to perform a meta-analysis. One limitation is that the original meta-analysis by Jores et al. (2019) included several low powered studies that differed on many levels (e.g., stimuli, study design) leading to increased heterogeneity which might have affected the estimation of the true effect sizes. However, the scope of the current article was to examine the practical implications using the SESOI and not to point out and possibly correct the limitations of the original meta-analysis. Nonetheless, future studies are necessary to provide better estimates of how alcohol influences witness testimony (see also Footnote 5).

Taken together, our analysis showed that alcohol intoxication exerts a statistically significant and practically-relevant effect on the reporting of correct details. Specifically, alcohol intoxication reduced the completeness of statements to a mean of about 3 details for both free and cued recall. Equally interesting, our analysis also demonstrated that although alcohol intoxication did not notably increase the reporting of incorrect details for free recall, it did so for cued recall. Collectively, then, our findings provide strong evidence in line with the idea that alcohol can statistically and practically significantly amplify the reporting of incorrect details and this amplification is of practical relevance.

## Concluding remarks

Many violent crimes are committed under the influence of alcohol (Kloft et al., 2021). When eyewitnesses, victims, or suspects need to talk about their crime-related experiences, it is essential to know whether their statements might have been adversely affected by alcohol intoxication. Psychologists are sometimes consulted to provide expert testimony in the courtroom on whether alcohol affects the validity of testimony. Expert witnesses will then commonly confer to the scientific literature on alcohol and memory and evaluate what the body of evidence says concerning the effect of alcohol on memory.

In a recent meta-analysis (Jores et al., 2019), although alcohol undermined the completeness of statements, it did not notably increase the reporting of incorrect details. This conclusion was based on the standardized effect sizes detected in the meta-analysis. In the current article, we make the case that relying on such standardized effect sizes is without meaning if they are not put into a broader context. Our position is that memory experts first need to decide and justify on what the smallest effect size of interest is in a given domain before the practical relevance of effect sizes can be discussed. Therefore, and in line with recent research (Riesthuis et al., 2022), we argued that in the realm of applied memory research, a decrease or increase of 1 detail could be seen as the smallest effect size of interest.

When this smallest effect size of interest was considered—and when we re-analyzed the data of the meta-analysis—we found that for both the reporting of correct and incorrect details, alcohol undermined the validity of testimony. When we specifically differentiated between free and cued recall, for correct details, we observed similar findings as when these memory tasks were combined (as they were in the meta-analysis). However, when we looked at the cued recall, alcohol intoxication significantly increased the reporting of incorrect details with a size thrice as large as our chosen smallest effect size of interest.

Our results imply that when the smallest effect size of interest is taken into account, alcohol intoxication increases the likelihood of invalid testimony, especially for cued recall. The findings about cued recall are especially important, given that interviews of witnesses, victims, and suspects by for example the police are not always in line with best practices. That is, such interviews oftentimes contain few open-ended questions to elicit free recall and possess more closed questions that might elicit cued recall (e.g., Lamb et al., 2007; Griffiths and Milne, 2018). Moreover, even before witnesses become ensnared in the criminal justice system, their memory reports might well arise from, or perhaps be contaminated by, discussions with others who unwittingly pose questions like those in cued-recall tasks.

Our analysis might also be helpful for memory experts working as expert witnesses in the courtroom. Specifically, our advice is that memory experts become acquainted with the concept of the smallest effect size of interest. The reason is that when the smallest effect size of interest is defined, justified, and used by such experts, expert witnesses could safely say that alcohol intoxication can negatively affect the validity of testimonies because it reduces the number of correct details and increases the reporting of incorrect details (and especially for cued recall). Second, although memory experts could use our analysis for their expert testimony, caution remains warranted. For example, we advise that where possible, memory experts working in cases should attempt to obtain as much detailed information about the case at hand such as how any formal interviews were conducted (e.g., were many closed questions used?). Only then, memory experts can provide more precise conclusions on how alcohol might have affected the validity of testimony. Third, when establishing *a priori* the smallest effect size of interest, researchers can use this to conduct equivalence tests. Equivalence tests provide helpful information on whether observed raw mean differences are equivalent or not to specified smallest effect sizes of interest. If they are not equivalent (as in our examples), they evince practical relevance.

We end with some recommendations and remarks for future directions in this area. First, we encourage discussion and future research addressing varying effect sizes of interest. We note that even if we had adopted a more liberal smallest effect sizes of interest, such as 2 or 3 details, our analysis would still have shown that alcohol intoxication negatively affects the validity of testimony and has practical relevance. But it is important to set these criteria *a priori*, and we believe it is imperative that memory scholars continue to discuss the choice for the smallest effect size of interest by, for example, digging into possible costs and benefits. Alternatively, to learn more about the smallest effect size of interest, it might be prudent to ask other populations such as judges, police officers, or jurors what they believe are the smallest effects of interest when it comes to the validity of witness evidence. Also, we encourage researchers to discuss smallest effect sizes of interest in other areas of applied memory research. To provide a concrete example, Nyman et al. (2021) discussed the importance of “serious errors”^8^ in person descriptions. According to them, “serious errors” increase the chance of arresting the wrong person. This idea of “serious errors” comes close to our view on the smallest effect size of interest.

Second, an important issue is whether the forgetting or misremembering of *any* detail is practically relevant. That is, one might contend that only details that are relevant for an investigation should be related to the smallest effect size of interest. For example, Oxburgh et al. (2012) noted that investigation relevant information is information about (i) what happened, (ii) how the crime was perpetrated, (iii) which persons were involved, (iv) when and where the crime happened, and (v) any items that were used during the crime.

However, sometimes, eyewitnesses forget/misremember a detail that is not strictly part of the definition of investigation relevant information and at first sight is not related to the investigation at all (e.g., the clothing of the offender). Nonetheless, even such details could potentially lead to egregious effects during a legal proceeding. For example, Granhag et al. (2013) showed that eyewitness descriptions of the murder of the Swedish foreign minister Anna Lindh frequently contained incorrect details. That is, 42% of reported attributes were incorrect such as the clothes of the offender. Although the reporting of such incorrect details might not be immediately relevant to an investigation, they might become relevant later as when for example a person comes to the police claiming to have seen an (innocent) person wearing these clothes.

Of course, our argument that any detail that is forgotten or misremembered might have negative effects in the courtroom is related to our cost–benefit analysis. Specifically, we postulate that any wrongful conviction should be prevented and thus any forgotten/misremembered detail should be of interest to legal professionals. Details might not be relevant for a case at the start of an investigation but may become relevant later on. Only including details that are immediately investigation relevant might exclude details that become important at a later stage. Again, researchers might adopt different smallest effect sizes of interest and provide different cost–benefit analyses. For example, researchers might argue that the re-victimization of real victims should always be prevented and hence, prefer more liberal smallest effect sizes of interest (such as 2, 3, or even more details). Of course, a challenge for such liberal thresholds is to make a solid case for why specifically a certain specified number of details would prevent the re-victimization of victims. Thus, different smallest effect sizes of interest might be chosen depending on the specific context such as the type of research and the to be used study paradigm, albeit that the choice for such effect sizes needs justification.

Nonetheless, a critical point here is that for the current article, we argued for the smallest effect size of interest of any detail that is forgotten or misremembered. However, an equal case can be made to situations in which *additional* details are remembered such as when witnesses or victims are interviewed using an empirically validated interview protocol such as the Cognitive Interview (Memon et al., 2010). Here too, one can posit that any detail that is *additionally* remembered could be regarded as the smallest effect size of interest. Such smallest effect size of interest is in the benefit of victims and might help in the prevention of re-traumatization. For example, when a victim additionally remembers that a culprit had a certain tattoo, then this might help the police in finding the perpetrator.

Third, our re-analysis focused on (1) the general effect of alcohol intoxication on the reporting of correct and incorrect details and (2) the effect of alcohol intoxication on the reporting of correct and incorrect details, separately for free and cued recall. Of course, many other possible mean differences might be interesting for a specific case, such as the details people recall when their sober versus intoxicated states match, or do not match, encoding. For example, in the original meta-analysis, it was shown that high levels of intoxication (BAC equal or above 0.10) exerted even larger memory undermining effects on correct details thereby being even more practically relevant. Also, when we examined the cued recall data concerning incorrect details, two studies included high levels of intoxication (van Oorsouw et al., 2012; Altman et al., 2018). When we examined the mean difference for these studies, a value of 3.68 (*SD* = 4.70) was detected showing also signs of practical relevance but based on only a very limited number of studies.^9^ This finding parallels recent work showing that with increasing levels of alcohol intoxication, participants were more susceptibility to the reporting of suggested false information (van Oorsouw et al., 2019; see below). Overall, our analysis is the first demonstration showing that alcohol affects the validity of testimony to a concerning degree. Though research into specific aspects related to alcohol and memory remains rather limited (e.g., effects of immediate versus delayed interviews on alcohol-memory effects), additional research is important to obtain more precise estimates about alcohol’s practical effects on memory.

Fourth and relatedly, we re-analyzed data from a recent meta-analysis on alcohol and memory (Jores et al., 2019). However, following this meta-analysis, recent studies have also focused on other aspects of relevance on how alcohol might affect memory. For example, van Oorsouw et al. (2019) showed that when improper interviewing practices were used (i.e., the presentation of suggestive information), alcohol increased levels of suggestibility and this finding was especially notable with rising alcohol levels. This result is in line with Mindthoff et al. (2021) demonstrating that low levels of alcohol intoxication did not affect the reporting of suggestive information. Furthermore, Jores et al.’s meta-analysis focused on the effect of alcohol intoxication on free and cued recall. Recent research has examined whether intoxicated people might benefit from being interviewed using empirically based interview protocols (e.g., Crossland et al., 2020). Crossland et al. (2020), for example, showed that although intoxicated participants did not additionally benefit from receiving a well-conducted interview, all participants (intoxicated or not) did have more complete statements when a well conducted interview was applied. Apart from Jores et al.’s meta-analysis, future meta-analyses might concentrate on other important practical aspects on how alcohol affects memory such as whether it might affect the reporting of suggested information or whether a Cognitive Interview might counteract the debilitating memory effects of alcohol (see also Bartlett et al., 2022).

Finally, although we stress the importance of establishing the smallest effect size of interest in applied memory research, another relevant aspect in expert witness testimony is inferential strength (Tullett, 2022). This concerns the issue whether scientific results are strong enough for drawing inferences about specific individuals or cases (Faigman et al., 2014; Tullett, 2022). For example, courts in the US have stipulated that expert testimony can be applied to individual cases when a “fit” exists between the expert testimony and the specific case (Faigman et al., 2014). That is, the rationale is that courts must decide whether, for example, research findings presented by expert witnesses are relevant to specific aspects in an individual case. So, this issue of “fit” is determined by courts themselves. However, we argue that apart from establishing the smallest effect size of interest, expert witnesses might also write in their reports whether certain research findings/characteristics (e.g., the studied sample) “fit” an individual case.

It is important to realize here that when, for example, expert witnesses discuss the smallest effect size of interest in the area of alcohol-memory research, this effect size can in itself not be applied to an individual case. Rather, when expert witnesses refer to certain research on alcohol and memory in their expert testimony, they should point out whether the findings from this research are practically relevant (based on a justified smallest effect size of interest). Additionally, they might discuss whether this body of research contains similar characteristics as in a certain individual case. For example, when the case is about an alcoholic and the expert witness highlights whether the included research was conducted with alcoholics.

To conclude, memory experts working as expert witnesses would increase the quality of their expert testimony when they involved the smallest effect size of interest in their work. We have demonstrated that when a specific smallest effect size of interest is selected, alcohol adversely affects the validity of testimonies by reducing the number of correct details and enhancing the reporting of incorrect details (during cued recall).

## Data availability statement

The data analyzed in this study is subject to the following licenses/restrictions: The original data can be requested from the authors of the meta-analysis on alcohol and memory (Jores et al., 2019). Requests to access these datasets should be directed to Heather Flower and for more information concerning the data sharing, please go to: https://osf.io/nrbvk/.

## Ethics statement

Ethical review and approval was not required for the study on human participants in accordance with the local legislation and institutional requirements. Written informed consent for participation was not required for this study in accordance with the national legislation and the institutional requirements.

## Author contributions

HO designed the study and study idea and wrote the first version. HO and PR were involved in the data analysis. All other co-authors provided comments on this version. All authors contributed to the article and approved the submitted version.

## Funding

The current manuscript has been supported by a C1 (No. C14/19/013) and FWO (No. G0D3621N) Research Project grant awarded to the HO.

## Conflict of interest

The authors declare that the research was conducted in the absence of any commercial or financial relationships that could be construed as a potential conflict of interest.

## Publisher’s note

All claims expressed in this article are solely those of the authors and do not necessarily represent those of their affiliated organizations, or those of the publisher, the editors and the reviewers. Any product that may be evaluated in this article, or claim that may be made by its manufacturer, is not guaranteed or endorsed by the publisher.

## Footnotes

^1^Although the word "reliability" is oftentimes used to refer to whether testimonies accurately reflect an experience, the correct term is "validity" (see also Arbiyah et al., 2021).

^2^This was based on an analysis across the following countries: Australia, the Netherlands, England and Wales, United States, and Indonesia.

^3^In such studies, researchers occasionally have listed several items of interest which they then compare with the testimonial performance of participants. This means that completeness is also related to the choice of which items were of interest to the researchers.

^4^Examples of cued recall questions are "What was the color of the car?," and examples of free recall questions are "Report everything."

^5^We received the data from Jores et al. (2019). We refer the reader to contact Jores et al. if they wish to look at the data as well. Important to note is that we were able to reproduce the main results as reported in Jores et al.

^6^When we split the data in free and cued recall, the numbers are quite similar (free recall: *M* = 3.03, *SD* = 10.97; cued recall: *M* = 3.10, *SD* = 6.21).

^7^We used this website to calculate the probability of superiority: rpsychologist.com in which a Cohen’s *d* or Hedges’ *g* can be inserted (Magnusson, 2022).

^8^Serious errors were seen as errors outside +/− 10 units (e.g., cm).

^9^Although there are only limited studies examining correct memory for peripheral and central details, we also explored mean differences between correct memory (free and cued recall) for such details as a function of intoxication. For peripheral details, the mean differences exceeded 1 and for cued recall this was almost 1 too (= 0.9). However, caution is warranted in interpreting these findings and future research is necessary.
